# Photo-Selective Nets and Pest Control: Searching Behavior of the Codling Moth Parasitoid *Mastrus ridens* (Hymenoptera: Ichneumonidae) under Varying Light Quantity and Quality Conditions

**DOI:** 10.3390/insects12070582

**Published:** 2021-06-28

**Authors:** María-José Yáñez-Díaz, Marcela Rodríguez, Selim Musleh, Luis Devotto, Gonzalo Silva, Eric Lucas

**Affiliations:** 1 Laboratorio de Entomología Aplicada, Departamento de Zoología, Facultad de Ciencias Naturales y Oceanográficas, Universidad de Concepción, Barrio Universitario s/n, Casilla 160-C, 4030000 Concepción, Chile; mayanez@udec.cl (M.-J.Y.-D.); semusleh@gmail.com (S.M.); 2 Núcleo Milenio de Salmónidos Invasores INVASAL, 4030000 Concepción, Chile; 3 Centro Tecnológico de Control Biológico, Centro Regional de Investigación Quilamapu, Instituto de Investigaciones Agropecuarias (INIA), Avda. Vicente Méndez 515, Casilla 426, 3780000 Chillán, Chile; ldevotto@gmail.com; 4 Laboratorio de Entomología, Departamento de Producción Vegetal, Facultad de Agronomía, Universidad de Concepción, Av. Vicente Méndez 595, Casilla 537, 3780000 Chillán, Chile; gosilva@udec.cl; 5 Laboratoire de Lutte Biologique, Département des Sciences Biologiques, Université du Québec à Montréal, 141 Avenue du Président-Kennedy, Montréal, QC H2X 1Y4, Canada; lucas.eric@uqam.ca

**Keywords:** biological control, *Cydia pomonella*, host localization, light quality, parasitism, photo-selective nets

## Abstract

**Simple Summary:**

Although substantial studies exists evaluating the effects of photo-selective nets (PSN) on physiological and morphological aspects of crops in different parts of the world, there is a paucity of studies regarding the effects of PSN on the searching behavior of natural enemies of insect pests. *Cydia pomonella* is a major insect pest of apples worldwide and the parasitoid *Mastrus ridens* is an important natural enemy of this pest. We evaluated the effects of pearl and red PSN and black standard nets (SN) on the searching behavior of this parasitoid. The host localization ability of the parasitoid was significantly affected by the net color. The females found their hosts faster under the pearl and red PSN compared to the black SN. However, no effects were found in terms of parasitism or the initial behavior of the parasitoid. Even though parasitism was not affected, host localization was delayed under black SN compared to the other PSN. Therefore, it is necessary to carry out further studies in field conditions to verify whether parasitism or other parameters could be affected.

**Abstract:**

Photo-selective nets (PSN) are used to manipulate the physiology of fruit crops. Besides their advantages to the crop, PSN potentially affect insect pests and their natural enemies. We aimed to assess the effects of these production systems on the searching behavior of the codling moth parasitoid, *Mastrus ridens*. We hypothesized that PSN and black standard nets (SN) affect the behavior of the parasitoid by delaying host localization and reducing parasitism. Laboratory experiments were carried out in closed cages under four treatment conditions: black SN, pearl PSN, red PSN, and no PSN as control (uncovered cages). Our results showed that the host localization of *M. ridens* was delayed under black SN and enhanced by pearl and red PSN. The PSN and the black SN did not affect the parasitism levels. In addition, the initial behavior of the parasitoid during the first 30 min of the bioassays was not affected by treatments. However, females spent most of the time walking around the arena, grooming, or resting, regardless of the color of the net. Parasitism was not affected under the PSN or the black SN; however, this must be verified in field conditions.

## 1. Introduction

The increased UV radiation observed after the weakening of the ozone layer and the increasing occurrence of unexpected climatic events (rain, hail, frosts, etc.) are challenging obstacles to global food production, particularly for fruits [1,2]. In response, crop production under protective structures such as nets is increasing [3,4]. Among other benefits, netting provides a reduction in the incidence of direct radiation by maintaining the suited temperature for a particular crop, leading to decreased fruit damage [5]. At present, black standard nets (SN) have been the most used by growers for bell peppers, cucumber, and tomato in countries such as Chile, Israel, and South Africa [6,7,8,9,10], mainly due to their low cost and because they provide a higher shading factor compared to other net colors [11,12]. Black SN reduce the amounts of PAR and UV light throughout the whole light spectra but do not affect the light quality [10]. In addition to light quantity, the light quality that passes through the nets onto the crops has been manipulated to promote certain physiological responses in target plants and to improve fruit quality. For this purpose, photo-selective nets (PSN) of different colors were developed to provide physical protection and differential filtration of the solar spectra. Different types of netting are used depending on the goals that the grower or the researcher wants to achieve [4,13,14]. Through the incorporation of light-reflective or chromatic elements into the plastic material, the modification of light spectra in the UV (200–400 nm), visible (400–700 nm), and red regions (600 nm and up) is possible [10,15]. For example, the pearl PSN reduce the light quantity (PAR, 400–700 nm) and at the same time affect the light quality, lowering the transmission of UVA and UVB light (280–400 nm) [10,16]. Compared to other PSN and black SN, under pearl PSN, there is a higher amount of diffuse light, which improves the light penetration into the inner, most shaded canopies of the crop [17]. This light manipulation provided by the pearl PSN increases the fruit size (apples and pears), plant productivity (peppers), and total yield (apples) [7,10,18]. Similar to the pearl PSN, the red PSN also enhance the light scattering throughout the crop, although to a lesser extent [10]. The red PSN are specifically designed to absorb three light bands (UV, blue, and green) and to enhance light transmission in the red and far-red ranges (600 nm and up) [10,15]. This light manipulation results in increased biomass (cucumber), vegetative growth (cucumber and foliage crops, e.g., *Pittosporum variegatum*), and average fruit weight (cucumber and pepper) [9,10].

At present, most of the studies on UV-absorbing nets and insect responses have focused on agricultural pests, including aphids, whiteflies, and mites [19,20,21]. Based on the available results, a general pattern has been raised in UV-deficient environments, showing negative effects in terms of both insect vision and orientation, lowering their flight and dispersion capability and delaying their arrival to the crop [20,22,23]. The knowledge of the effects of the PSN on the activity of natural enemies is very limited. One of the few studies related to parasitoids in greenhouse trials showed that by using UV-absorbing plastic sheets, some hymenopteran parasitoids were not affected in terms of their ability to locate hosts [24]. However, these authors stated that to some extent, other parasitoids such as *Eretmocerus mundus* used UV light for long-range host location; therefore, their host searching efficiency was affected by changes in the amount of UV light. It has been proposed that parasitoids that were not affected by reduced UV light used mainly olfactory signals instead of visual cues for host finding, while species with poor performance under UV-deficient environments used mainly visual stimuli for host finding [20,24].

In the present study, to assess the effects of the black SN, the pearl PSN, and the red PSN on the searching behavior of a biological control agent, we selected the codling moth *Cydia pomonella* (L.) (Lepidoptera: Tortricidae) and its natural enemy *Mastrus ridens* Horstmann (Hymenoptera: Ichneumonidae). Apple is one of the most important fruit crops in Chile, and netting is widely used in apple cultivars, especially in Fuji and related varieties. The PSN and the black SN used in this study were chosen because (i) the black SN is commonly used by growers in Chile and (ii) the pearl and red PSN were recently introduced for crop protection in Chile [25]. *C. pomonella* is the main pest insect of apple, pear, and walnut plants worldwide [26]. The area of origin of *C. pomonella* is Eurasia and it is present in all main temperate fruit growing areas of the world [27]. Because *C. pomonella* larvae cause direct damage to fruit, control measures rely heavily on frequent insecticide sprays, which is against consumer preference and increases the risk of insecticide resistance [28,29,30]. To reduce the use of insecticides, several natural enemies have been evaluated for *C. pomonella* control, including pathogens, predators, and parasitoids [31]. Early in the 1990s, both human society and science benefited from the end of the Cold War, as US researchers were granted access to the center of origin of *C. pomonella* in the former USSR territory. Several hymenopteran parasitoids were found and some were brought to the US for further evaluation, including *M. ridens*, a gregarious idiobiont ectoparasitoid of fifth-instar larvae or pre-pupal *C. pomonella* [32]. Later, this parasitoid was introduced to Chile and Argentina under a classical biological control approach [33,34,35].

For some hymenopteran parasitoids, phototactic activity has been positively associated with light quantity, as reflected in higher parasitism levels [36,37]. Additionally, the visual system used for hymenopteran species is mainly composed of blue, green, and UV photoreceptors, meaning the parasitoids respond to different wavelengths, showing preferences for certain colors and light intensities [36,38,39]. The host searching efficiency of *M. ridens* in netted orchards has not been studied in any of the countries where the parasitoid has been introduced. Therefore, our research aimed to assess the effects of the PSN and black SN on the searching behavior of the parasitoid *M. ridens* in laboratory conditions. Specifically, we measured the time needed by *M. ridens* for host localization and compared the parasitism levels under the PSN and black SN. We hypothesized that the presence of the PSN or black SN affects the behavior of *M. ridens* by delaying host localization and reducing parasitism on fifth-instar *C. pomonella* larvae. We expected that the lower light levels and different light spectra under PSN and black SN, compared to no PSN, would diminish the searching behavior of *M. ridens*.

## 2. Materials and Methods

Laboratory bioassays were carried out to assess the effects of the PSN and black SN on the host searching behavior of *M. ridens*; in particular, to assess whether the PSN and black SN influence the host finding capability and parasitism levels.

### 2.1. Insects

To conduct the bioassays, the chosen hosts were fifth-instar *C. pomonella* larvae for the ectoparasitoid *M. ridens*. *M. ridens* females are attracted to kairomones emitted by the cocoons to locate potential hosts [40]; therefore, we offered four fifth-instar *C. pomonella* larvae that had recently spun cocoons (≤24 h) to a single female. Both fifth-instar *C. pomonella* larvae and *M. ridens* pupae were obtained from colonies maintained in the Entomology Laboratory, Center for Biological Control Technology, INIA Quilamapu, (Chillán, Chile). The parasitoid was imported from the United States in 2004 and subsequently maintained for sixteen years. The colony of *C. pomonella* has been maintained for over fourteen years, with wild individuals added periodically, mainly from apple orchards [33]. *C. pomonella* eggs and larvae were reared on round plastic containers (8 cm high and 12 cm diameter) with 80–100 mL of agar-based diet. Both *C. pomonella* and *M. ridens* were brought to the Applied Entomology Laboratory, Universidad de Concepción, and maintained at 26 °C with a 16:8 (L/D) photoperiod in a growing chamber. *M. ridens* adults that emerged from the pupae were kept in wood and glass cages (31.5 × 21.5 × 23 cm) and provided with honey and water ad libitum.

### 2.2. Host Localization

To study the effects of the PSN and black SN treatments on the host localization ability of *M. ridens*, bioassays were conducted inside an experimental unit, consisting of a 12 L plastic box (22.6 × 23 × 20.5 cm for high, length, and width, respectively) built using two 6 L plastic boxes attached with clay, one inverted on top of the other. Each cage had holes on the side covered with transparent muslin to allow ventilation inside the cage. For all bioassays, four treatments were used: black SN, pearl PSN, and red PSN above the cage, while no PSN was used as control (uncovered cages) (Table 1). The PSN and the black SN were put directly above each cage to modify the quantity and quality of light inside the experimental unit. Inside each cage were six apple plants (~10–15 cm height) placed on top of a green foam base inside a plastic pot. As a light source, two 36 W tubes (SERA Daylight Brilliant 36 W 60 K T8) were located 57 cm above the surface of the cage. One *M. ridens* couple, consisting of one male and one female (aged 24–48 h), was used alongside four fifth-instar *C. pomonella* larvae on individual cardboard pieces, based on the attack rate registered by this parasitoid (1–4 hosts/day in the laboratory) [41]. The day before the bioassays, the fifth-instar *C. pomonella* larvae were put individually on cardboard pieces to allow them to spin their cocoon. *M. ridens* adults were fed ad libitum with honey and water before and during the bioassays. In one plant at one extreme of the cage, four fifth-instar *C. pomonella* larvae were attached to the trunk of the plant with a double-sided adhesive. The *M. ridens* couple was introduced by the ventilation hole at the other extreme of the cage. Each treatment was replicated 15 times. The experiments were carried out at 27 ± 1 °C, with a 16:8 (L/D) photoperiod and 60% RH. To assess the effects of the PSN, black SN, and no PSN on the host localization capability of the parasitoid, the time spent finding the host was recorded every 30 min for six hours and at 24 h after the beginning of the bioassays.

### 2.3. Parasitism

We tested if the PSN and the black SN affect the parasitism activity of *M. ridens*. For this purpose, the *M. ridens* couple and the four fifth-instar *C. pomonella* larvae were introduced in the same experimental unit described in Section 2.2. After 24 h of exposure to the female *M. ridens*, the cardboard pieces containing the fifth-instar *C. pomonella* larvae were placed on a Petri dish (5 cm diameter) on a growing chamber at 26 °C with a 16:8 (L/D) photoperiod until adult emergence. Each treatment was replicated 15 times. We recorded the parasitism level corresponding to the number of parasitized larvae by female out of the four larvae offered. To assess the natural mortality of *C. pomonella* larvae, we placed four fifth-instar larvae on individual cardboard pieces on a Petri dish. This was replicated 7 times (each time we carried out a set of parasitism bioassays). After 24 h, we moved them to a growing chamber under the same conditions established in Section 2.3 until adult emergence. As the mortality of *C. pomonella* larvae on the Petri dishes was low, we did not correct the parasitism data in the analysis. Additionally, we measured other parameters related to parasitism, such as the female fertility (total parasitoid larvae attached to a host/day), the achieved fecundity (total emerged adults from a female/day), and the sex ratio (percentage of female offspring respect to the total adult offspring). Regarding the fertility data, we counted the number of larvae laid by a female instead of counting the number of eggs laid in order to avoid unnecessary manipulation and risk of mortality for the eggs due to the handling process. The latter data are presented as supplementary materials (see Section 3.2).

### 2.4. Parasitoid Behavior

We assessed the effects of the PSN and black SN on the behavior of the female parasitoids during the first 30 min after their introduction to the experimental unit. These observations were used to describe the parasitoid responses to the experimental unit. Parasitoid behavior was cataloged based on behavioral categories modified from Charles et al. [42]. The parasitoid responses were categorized into two phases. Phase 1 (localization and examination) consisted of the (1.1) pre-contact (walking aimlessly around the arena, grooming, or resting), (1.2) mating (copulation behavior between male and female), (1.3) examination (walking around the host, with a continuous examination by antennae and ovipositor), and (1.4) acceptance (host acceptance and stinging, terebra insertion) sub-phases. Phase 2 of our observations (pre-contact) focused on the pre-contact sub-phase described in Phase 1. Phase 2 was divided into two sub-phases, (2.1) searching (walking around the arena with continuous antennation (the antennae are extended)) and (2.2) grooming (resting but moving antennae, legs, abdomen, or ovipositor). Observations of parasitoid behavior were replicated 15 times under each treatment.

### 2.5. Data Analysis

To determine if the PSN and black SN influence the time required by *M. ridens* females to find the fifth-instar *C. pomonella* larvae, a generalized linear model (glm) with binomial distribution was performed using the statistical package glmm TMB [43]. For the analysis, the dependent variable was whether the parasitoid located the fifth-instar *C. pomonella* larvae at each given time (binary data: 1 when the parasitoid found the host; 0 when the parasitoid did not find the host), while the independent variables were the four different treatments and the 13 time intervals. To determine whether the PSN and black SN treatments had significant effects on the parasitism and because the data were not normally distributed, a non-parametric Kruskal–Wallis test for multiple comparisons was performed. Finally, to test whether the PSN and black SN influenced the time allocated by the females to different behaviors (phases 1 and 2, described in Section 2.4), and since the data were not normally distributed, a non-parametric Kruskal–Wallis test was performed. Additionally, to evaluate if there were significant differences within each treatment for the time spent by females on each behavior sub-phase (phase 1 and 2), a Wilcoxon signed-rank test was used for paired observations. All analyses were carried out in R v. 3.6.0 [44].

## 3. Results

### 3.1. Effects of the PSN and Black SN on the Host Localization Capability of M. ridens

**Figure 1:**
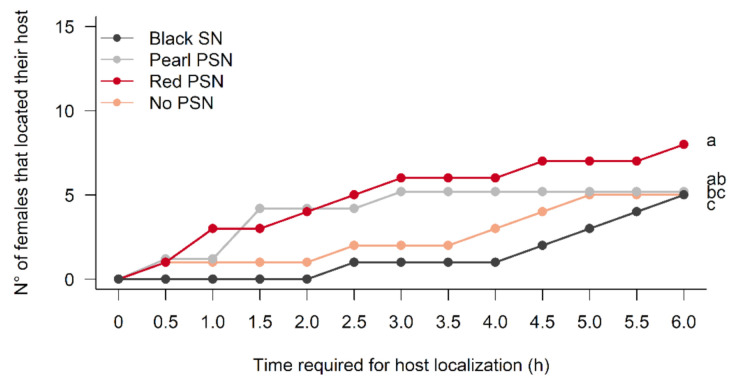
Accumulated number of *M. ridens* females that located the fifth-instar *C. pomonella* larvae under the PSN and black SN within the first six hours of observation. Different letters next to each line indicate significant differences among treatments according to the glm test. The significance of each letter is as follows: red PSN (a), pearl PSN (ab), no PSN (bc), and black PSN (c).

The host localization capability levels of *M. ridens* differed significantly depending on the use of colored PSN or black SN in our experimental cages (Figure 1, Table S1). Under the black SN, *M ridens* host localization was delayed compared to the other treatments, being significantly different from the pearl PSN (df = 775, *p* = 0.0003) and the red PSN (df = 775, *p* = 0.0001). However, no significant differences were observed along the observation time between the host localization capabilities of the parasitoids under the black SN compared to parasitoids under no PSN (df = 775, *p* = 0.072). The observed response of the parasitoids under the pearl PSN was similar to the parasitoids under the red PSN (df = 775, *p* = 0.0750) and no PSN (df = 775, *p* = 0.052). Under the red PSN, the host localization capability by females was enhanced, with a higher proportion of females reaching the host compared to the control (df = 775, *p* = 0.0003). At 24 h after the bioassay started, there were no significant differences in the host localization among treatments (Table S2).

### 3.2. Effects of the PSN and Black SN on the Parasitism Level of M. ridens

**Figure 2:**
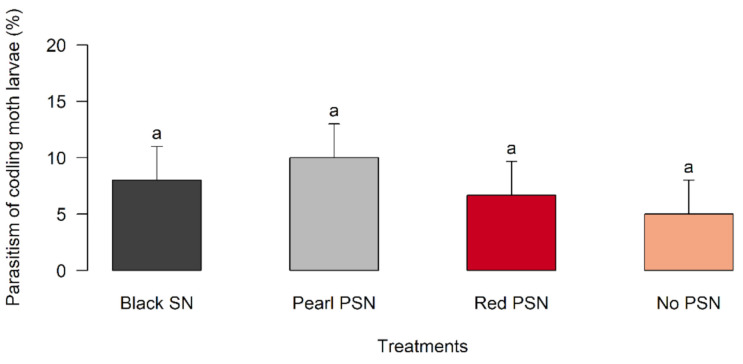
Effects of the PSN and black SN on the parasitism (%) (mean ± SE, *n* = 15) of *M. ridens* on fifth-instar *C. pomonella* larvae. The same letters above bars and SE indicate no significant differences among treatments according to the Kruskal–Wallis test.

A Kruskal–Wallis analysis of the parasitism percentage revealed that the color of the nets did not affect the parasitism level of the parasitoid females in laboratory conditions (Figure 2, Table S3). Larval parasitism ranged from 5% to 10% depending on the color of the nets but no significant differences were detected (df = 3, *p* = 0.668). Furthermore, we found that related parameters such as fertility, realized fecundity, and offspring sex ratio were not affected by the use of nets (fertility: df = 3, *p* = 0.702; realized fecundity: df = 3, *p* = 0.769; offspring sex ratio: df = 3, *p* = 0.944) (Table S3).

### 3.3. Effect of the PSN and the Black SN on the Behavior of M. ridens Females

**Figure 3:**
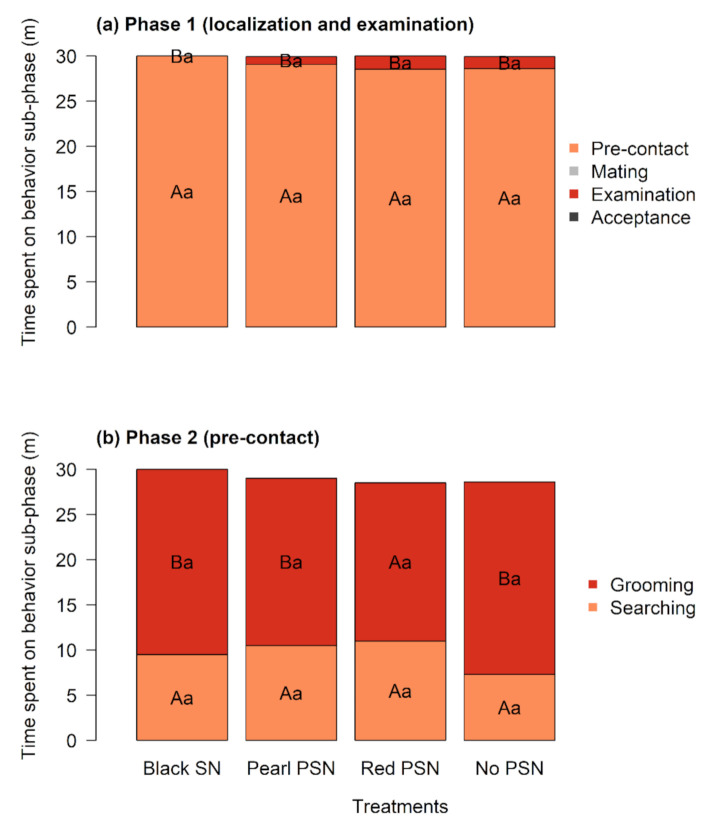
Effects of the PSN and black SN on the mean time (minutes) allocated by *M. ridens* females to the different behavior categories: (**a**) phase 1 (localization and examination): (1.1) pre-contact, (1.2) mating, (1.3) examination, and (1.4) acceptance *; (**b**) phase 2 (pre-contact): (2.1) searching and (2.2) grooming. The total observation time was 30 min. Different lowercase letters in bars indicate significant differences among treatments for each sub-phase according to the Kruskal–Wallis test. Uppercase letters in bars indicate significant differences among sub-phases within each treatment according to the Wilcoxon signed-rank test for paired observations. Time spent by *M. ridens* females on the mating sub-phases and acceptance of phase 1 were so low that they are not shown in the figure.

We observed the behavior of *M. ridens* females to assess the effects of the PSN and black SN during the first 30 min immediately after their release inside the experimental units. We found no significant differences in the time spent on phase 1 of behavior among treatments (Figure 3a, Table S4). Regardless of the color of the net, all females spent most of their time in the pre-contact sub-phase (df = 3, *p* = 0.523), which accounted for more than 95% of the total time among treatments, especially under the black SN, where the females spent the whole 30 min of observation on this behavior sub-phase. The time spent on pre-contact was significantly higher than the time spent on the other three behavior sub-phases under all four treatments (V = 120, *p* < 0.05). The analysis of mating also showed no differences among treatments (df = 3, *p* = 0.565). It is important to state that we only observed mating under the pearl PSN and no PSN. The mating between the male and the female lasted no longer than one minute, and after termination the male moved away from the female. The female stayed still for a few seconds before resuming the host search. The examination process, whereby the female finds the host and taps the cardboard with their antennae, was a very rare event and was not affected by the treatments (df = 3, *p* = 0.792). No hosts were reached under the black SN. A single female reached the host during the first 30 min under the pearl PSN, the red PSN, and no PSN. Although some females reached the host during this time, acceptance was not registered in any of the four treatments tested.

Furthermore, for phase 2 behavior (Figure 3b, Table S5), the results showed no differences among treatments for searching (df = 3, *p* = 0.301) and grooming (df = 3, *p* = 0.382). In terms of the behavior within each treatment, except under the red PSN (V = 32, *p* = 0.117), the time spent on grooming was significantly higher than the time spent searching (black SN: V = 12.5, *p* = 0.007; pearl PSN: V = 19, *p* = 0.038; no PSN: V = 4.5, *p* = 0.003).

## 4. Discussion

For effective parasitism, the parasitoid first must find the environment where the potential host is and then properly locate the host for reproduction [45]. We were interested in particular in the visual stimuli that are relevant for the process of host finding [46,47]. The PSN and black SN can modify the light quantity and quality and potentially interfere with the behavior of both pests and parasitoids [24]. Our results indicated that under the black SN, host localization was delayed, supporting our hypothesis about the negative effects on the host finding capabilities of the parasitoid in low-light conditions. This should be considered whenever this net is used simultaneously with biological control agents in the field. The highest host localization rates occurred under the pearl and red PSN. The pearl and red PSN increased the amount of diffused light in the environment compared to the other treatments, improving the light penetration inside the canopy [10]. Based on our host localization results, we suggest that a higher light availability enhances the host finding capability of *M. ridens*. Although visual cues are important in host finding, chemical cues associated with the host also play a major role in host localization [48]. Under our experimental conditions, we could not determine the extent to which visual or chemical cues influenced the host localization ability of *M. ridens* because the success of locating a host depends on multiple mechanisms [46,48], which we could not separate. Other ichneumonid parasitoids use antennal tapping, olfaction, and visual contrast for host localization [49,50]. According to Chiel et al. and Chyzik et al. [20,24], the parasitoids *Aphidius colemani* Viereck (Hymenoptera: Braconidae), *Diglyphus isaea* Walker (Hymenoptera: Eulophidae), and *Aphidius matricariae* (Haliday) (Hymenoptera: Braconidae) were not affected in terms of host localization capability under UV-filtered environments, probably because these parasitoids use different cues for host localization or because their visual capabilities are not significantly affected under UV-deficient light. Nevertheless, our results showed that during the first six hours of the bioassays, the capability of the parasitoid to find a host was delayed under the black SN, suggesting that during the initial host finding process, the visual stimuli are relevant for *M. ridens*.

The parasitism, fecundity, and sex ratio are some of the most relevant factors when rearing parasitoid wasps [51]. Our results showed that the PSN and black SN did not have significant effects on the parasitism. This suggest that under our experimental setup, the light quantity and quality are not the main factors influencing parasitism. It has been established that in laboratory conditions, the parasitoid attacks a maximum of four hosts a day [41]. In our study, *M. ridens* females did not parasitize more than one host per day. A similar result was observed by Hougardy et al. [52], who also found that *M. ridens* individuals parasitize just one or two hosts a day.

In terms of the female behavior during the first 30 min, our results showed that for phase 1 of the observations (pre-contact, mating, examination, acceptance), the PSN and black SN did not influence the time spent by the female on each sub-phase. The fact that there were no differences in the time spent on each sub-phase shows that light quantity and quality do not significantly influence the time allocation decisions made by a female, at least during the first 30 min. Once the female and host are in the same habitat, the searching behavior is accomplished by walking, oriented by physical, visual, and olfactory cues [47,48]. The latter can guide the flight of the parasitoid to the habitat where the host is located (long-range cues), a well-known fact in the attraction of *M. ridens* to *C. pomonella* [40]. Therefore, the volume of our experimental units and the enclosed environment could have saturated the cage with cues, causing the female to spend similar times on each sub-phase, regardless of the color of the net. However, under all four treatments, the time spent on the pre-contact sub-phase was significantly higher than the other three sub-phases. In phase 2, the pre-contact behavior is dominated by walking around the experimental cage with continuous antennae movement, sporadically interrupted by grooming or resting [42]. Indeed, we observed all of these behaviors inside the experimental unit, with the time spent grooming being significantly higher than the times spent searching and walking around the cage, except under the red PSN environment, where the times between these sub-phases were similar. The females possibly spent more time grooming (moving antennae, abdomen, and ovipositor) to detect certain chemical cues that help them orient towards the *C. pomonella* cocoons [40]. Even though some females were observed examining the host on the cardboard within the first 30 min, the acceptance behavior was never registered under any treatment. It is possible that under our experimental conditions, the parasitoid needed more time to find and accept the host.

The PSN and black SN have been used as protection tools and as a crop production methods worldwide, with the majority of studies having focused on their influence on plant physiology and fruit quality [10,53,54,55]. We must consider that the utilization of PSN and black SN can also influence insect pests and their natural enemies [56]. Overall, our results show that under laboratory conditions, the PSN and black SN affect the host localization ability of *M. ridens* but do not influence the parasitism. As mentioned earlier, there are contrasting results in the literature regarding host localization; it has been proposed that the host finding capability is not influenced by the presence of the PSN, with host–plant signals and olfactory cues rather than visual cues being the main drivers for host finding [20,57]. On the other hand, some hymenopteran parasitoids species have difficulty locating their host under UV light deficiency conditions, preferring unfiltered light [24,58]. In this sense, we agree with the statements made by Legarrea et al. and Chiel et al. [24,56], who suggested that the responses of parasitoids to UV light deficiency are species-specific; thus, the result of the utilization of PSN on the host finding capability should not be generalized to every species. Therefore, additional studies should be carried out to address specific parasitoid–host interactions under photo-selective nets. Our results indicate that the utilization of PSN, specifically the pearl and red PSN, is compatible with the activity of the parasitoid *M. ridens*. Nevertheless, studies in natural conditions are also necessary to verify whether host localization is also affected in the field as it was in the laboratory microenvironment.

## 5. Conclusions

The fitness of parasitoids can be determined by several factors, with reproduction and host location capability being some of the most important. The results of our study showed that the host localization ability of *M. ridens* was enhanced by the pearl and red PSN and delayed under the black SN; however, the parasitism was not affected by the PSN or black SN. The use of PSN (pearl and red) and biological control agents are compatible strategies that can be included in integrated pest management (IPM) programs. Both strategies can be involved in reducing pest populations, as well as reducing the use of insecticides in agro-ecosystems. Even though parasitism was not affected, host localization was delayed under black SN compared to the other PSN; therefore, it is necessary to carry out further studies in field conditions to verify whether parasitism or other parameters could potentially be affected under these production systems.

## Figures and Tables

**Table 1:** Light intensity was measured as the photosynthetic active radiation (PAR) and shading factor provided by the photo-selective and black standard nets (PSN and SN, respectively) used in the experiments.

PSN Color	Supplier	Specifications	PAR (µmol m^−2^ s^−1^)	Shading Factor (%)
No PSN (control)	-	-	7	0
Pearl PSN	ChromatiNet^®^, Polysack Industries, Negev, Israel	HDPE monofilament, mesh size 5 × 2 mm	6	20
Red PSN	ChromatiNet^®^, Polysack Industries, Negev, Israel	HDPE monofilament, mesh size 5 × 2 mm	5	20
Black SN	ChromatiNet^®^, Polysack Industries, Negev, Israel	Polyethylene, mesh size 3 × 1 mm	2	80

## Data Availability

Data are contained within the article and its supplementary materials.

## Supplementary Materials

The following are available online at https://www.mdpi.com/article/10.3390/insects12070582/s1: Table S1: Accumulated proportion of *Mastrus ridens* females that found the *Cydia pomonella* larvae during all time intervals observed (*n* = 15; mean ± SE). Table S2: Host localization (*p* values) among treatments at 24 h after the bioassay started according to the generalized linear model (glm) test. Table S3: Biological parameters of *Mastrus ridens* females (*n* = 15 (except for sex ratio), mean ± SE) under the four treatments. *Number in parenthesis corresponds to the total number of adults who emerged. Different lowercase letters in columns next to means ± SE indicate significant differences among treatments for each of the biological parameters according to the Kruskal–Wallis test. Table S4: Time (m) (mean ± SE) allocated by *M. ridens* females to the behavior sub-phases from phase 1, modified from the study by Charles et al. (2013). The total observation time was 30 min. Different lowercase letters in columns next to means ± SE indicate significant differences among treatments in the time spent on each sub-phase according to the Kruskal–Wallis test. Different uppercase letters in rows indicate significant differences among the time spent on each sub-phase within each treatment according to the Wilcoxon signed-rank test for paired observations. Table S5: Time (m) (mean ± SE) allocated by *M. ridens* females to the behavior sub-phases from phase 2, modified from the study by Charles et al. (2013). Different lowercase letters in columns next to means ± SE indicate significant differences among treatments in the time spent on each sub-phase according to the Kruskal–Wallis test. Different uppercase letters in rows indicate significant differences among the time spent on each sub-phase within each treatment according to the Wilcoxon signed-rank test for paired observations.
