# Supplementary material for: Photo-Selective Nets and Pest Control: Searching Behavior of the Codling Moth Parasitoid Mastrus ridens (Hymenoptera: Ichneumonidae) under Varying Light Quantity and Quality Conditions
Source: Insects. 2021 Jun 28;12(7):582. doi: 10.3390/insects12070582 (PMC8305221; doi:10.3390/insects12070582)
Supplement: Supplementary file 1 [file insects-12-00582-s001.zip › Table S1.pdf]

Table S1. Accumulated proportion of females *Mastrus ridens* that found the *Cydia pomonella* larvae during all time intervals observed (*n*=15; mean ± SE).

Treatments	Time after the introduction of the parasitoid inside the experimental cage (h)												
	**0.5**	**1.0**	**1.5**	**2.0**	**2.5**	**3.0**	**3.5**	**4.0**	**4.5**	**5.0**	**5.5**	**6.0**	**24.0**
No PSN (control)	0.07 ± 0.07	0.07 ± 0.07	0.07 ± 0.07	0.07 ± 0.07	0.13 ± 0.09	0.13 ± 0.09	0.13 ± 0.09	0.20 ± 0.11	0.27 ± 0.12	0.33 ± 0.13	0.33 ± 0.13	0.40 ± 0.13	0.47 ± 0.13
Pearl PSN	0.07 ± 0.07	0.07 ± 0.07	0.27 ± 0.12	0.27 ± 0.12	0.27 ± 0.12	0.33 ± 0.13	0.33 ± 0.13	0.33 ± 0.13	0.33 ± 0.13	0.33 ± 0.13	0.33 ± 0.13	0.33 ± 0.13	0.47 ± 0.13
Red PSN	0.07 ± 0.07	0.20 ± 0.11	0.20 ± 0.11	0.27 ± 0.12	0.33 ± 0.13	0.40 ± 0.13	0.40 ± 0.13	0.40 ± 0.13	0.47 ± 0.13	0.47 ± 0.13	0.47 ± 0.13	0.53 ± 0.13	0.60 ± 0.13
Black SN	0.00 ± 0.00	0.00 ± 0.00	0.00 ± 0.00	0.00 ± 0.00	0.07 ± 0.07	0.07 ± 0.07	0.07 ± 0.07	0.07 ± 0.07	0.13 ± 0.09	0.20 ± 0.11	0.27 ± 0.12	0.33 ± 0.13	0.60 ± 0.13
