# Supplementary material for: Photo-Selective Nets and Pest Control: Searching Behavior of the Codling Moth Parasitoid Mastrus ridens (Hymenoptera: Ichneumonidae) under Varying Light Quantity and Quality Conditions
Source: Insects. 2021 Jun 28;12(7):582. doi: 10.3390/insects12070582 (PMC8305221; doi:10.3390/insects12070582)
Supplement: Supplementary file 1 [file insects-12-00582-s001.zip › Table S2.pdf]

Table S2. Host localization (p values) among treatments at 24 hours after the bioassay started according to the generalized linear model (glm) test.

Treatments	No PSN (control)	Pearl PSN	Red PSN
Pearl PSN	1.00	-	-
Red PSN	0.46	0.46	-
Black SN	0.46	0.46	1.00
