# Supplementary material for: Photo-Selective Nets and Pest Control: Searching Behavior of the Codling Moth Parasitoid Mastrus ridens (Hymenoptera: Ichneumonidae) under Varying Light Quantity and Quality Conditions
Source: Insects. 2021 Jun 28;12(7):582. doi: 10.3390/insects12070582 (PMC8305221; doi:10.3390/insects12070582)
Supplement: Supplementary file 1 [file insects-12-00582-s001.zip › Table S3.pdf]

Table S3. Biological parameters of female *Mastrus ridens* (*n*=15 (except for sex ratio), mean ± SE) under the four treatments tested. *Number in parenthesis corresponds to the total number of adults who emerged. Different lowercase letters in columns next to mean ± SE indicate significant differences among treatments for each of the biological parameters according to the Kruskal-Wallis test.

Treatments	Biological parameters			
	Parasitism (%)	Fertility (number of larvae hatched)	Realized fecundity (number of adults emerged)	Sex ratio* (% of females in relation to total adults emerged)
No PSN (control)	5 ± 0.03a	0.9 ± 0.5a	0.7 ± 0.4a	30 (n = 10)a
Pearl PSN	10 ± 0.03a	1.0 ± 0.4a	0.8 ± 0.3a	33 (n = 12)a
Red PSN	7 ± 0.03a	0.7 ± 0.4a	0.5 ± 0.3a	25 (n = 8)a
Black SN	8 ± 0.03a	1.4 ± 0.5a	0.8 ± 0.4a	17 (n = 12)a
