# Supplementary material for: Photo-Selective Nets and Pest Control: Searching Behavior of the Codling Moth Parasitoid Mastrus ridens (Hymenoptera: Ichneumonidae) under Varying Light Quantity and Quality Conditions
Source: Insects. 2021 Jun 28;12(7):582. doi: 10.3390/insects12070582 (PMC8305221; doi:10.3390/insects12070582)
Supplement: Supplementary file 1 [file insects-12-00582-s001.zip › Table S4.pdf]

Table S4. Time (m) (mean ± SE) allocated by *M. ridens* females to the behavior sub-phases from phase 1 modified from Charles et al. (2013). The total observation time was 30 minutes. Different lowercase letters in columns next to mean ± SE indicate significant differences among treatments in the time spent on each sub-phase according to the Kruskal-Wallis test. Different uppercase letters in rows indicate significant differences among the time spent on each sub-phase within each treatment according to the Wilcoxon Signed-Rank test for paired observations.

**Treatments**	**Behaviors**			
	**Pre-contact**	**Mating**	**Examination**	**Acceptance**
No PSN (control)	28.60 ± 1.33 aA	0.07 ± 0.07 aB	1.33 ± 1.33 aB	0.00 ± 0.00 aB
Pearl PSN	29.07 ± 0.86 aA	0.07 ± 0.07 aB	0.87 ± 0.87 aB	0.00 ± 0.00 aB
Red PSN	28.53 ± 1.47 aA	0.00 ± 0.00 aB	1.47 ± 1.47 aB	0.00 ± 0.00 aB
Black SN	30.00 ± 0.00 aA	0.00 ± 0.00 aB	0.00 ± 0.00 aB	0.00 ± 0.00 aB
