# Supplementary material for: Photo-Selective Nets and Pest Control: Searching Behavior of the Codling Moth Parasitoid Mastrus ridens (Hymenoptera: Ichneumonidae) under Varying Light Quantity and Quality Conditions
Source: Insects. 2021 Jun 28;12(7):582. doi: 10.3390/insects12070582 (PMC8305221; doi:10.3390/insects12070582)
Supplement: Supplementary file 1 [file insects-12-00582-s001.zip › Table S5.pdf]

Table S5. Time (m) (mean ± SE) allocated by *M. ridens* females to the behavior sub-phases from phase 2 modified from Charles et al. (2013). Different lowercase letters in columns next to mean ± SE indicate significant differences among treatments in the time spent on each sub-phase according to the Kruskal-Wallis test. Different uppercase letters in rows indicate significant differences among the time spent on each sub-phase within each treatment according to the Wilcoxon Signed-Rank test for paired observations.

**Treatments**	**Behaviors**	
	**Searching**	**Grooming**
No PSN (control)	7.33 ± 1.30 aA	21.27 ± 1.81 aB
Pearl PSN	10.47 ± 1.63 aA	18.53 ± 1.72 aB
Red PSN	11.00 ± 1.93 aA	17.53 ± 2.25 aA
Black SN	9.53 ± 1.48 aA	20.47 ± 1.48 aB
